# Genomic characterization of a reemerging Chikungunya outbreak in Kedougou, Southeastern Senegal, 2023

**DOI:** 10.1080/22221751.2024.2373308

**Published:** 2024-06-27

**Authors:** Idrissa Dieng, Bacary Djilocalisse Sadio, Alioune Gaye, Samba Niang Sagne, Marie Henriette Dior Ndione, Mouhamed Kane, Mamadou Korka Diallo, Bocar Sow, Safietou Sankhe, Ousseynou Sene, Amadou Diallo, Madeleine Dieng, Serge Freddy Moukaha Doukanda, Maimouna Mbanne, Seynabou Mbaye Ba Souna Diop, Diamilatou Balde, Mignane Ndiaye, Khalidou Djibril Sow, Maryam Diarra, Abdoulaye Sam, Ababacar Mbaye, Boubacar Diallo, Yoro Sall, Ousmane Faye, Boly Diop, Abdourahmane Sow, Amadou Alpha Sall, Cheikh Loucoubar, Ndongo Dia, Oumar Faye, Diawo Diallo, Gamou Fall, Scott C. Weaver, Mamadou Aliou Barry, Mawlouth Diallo, Moussa Moise Diagne

**Affiliations:** a Virology Department, Institut Pasteur de Dakar, Dakar, Senegal; b Zoology Medical Department, Institut Pasteur de Dakar, Dakar, Senegal; c Epidemiology, Clinical Research and Data Science Department, Institut Pasteur de Dakar, Dakar, Senegal; d Health Emergency Operations Center, Ministry of Health, Dakar, Senegal; e Prevention Department, Ministry of Health, Dakar, Senegal; f Kedougou Medical Region, Ministry of Health, Kedougou, Senegal; g Public Health Direction, Institut Pasteur de Dakar, Dakar, Senegal; h Department of Public Health and Preventive Medicine, Faculty of Medicine, Pharmacy and Odonto-stomatology, Université Cheikh Anta Diop de Dakar, Dakar, Senegal; i World Reference Center for Emerging Viruses and Arboviruses, Institute for Human Infections and Immunity and Department of Microbiology and Immunology, University of Texas Medical Branch, Galveston, TX, USA

**Keywords:** Chikungunya, Kedougou, Southeastern Senegal, outbreak, genomic characterization

## Abstract

Chikungunya virus has caused millions of cases worldwide over the past 20 years, with recent outbreaks in Kedougou region in the southeastern Senegal, West Africa. Genomic characterization highlights that an ongoing epidemic in Kedougou in 2023 is not due to an introduction event but caused by the re-emergence of an endemic strain evolving linearly in a sylvatic context.

## Introduction

Chikungunya virus (CHIKV) is a mosquito-borne virus that has caused significant epidemics over the past 20 years with millions of cases reported worldwide. CHIKV is classified into West African (WA), East-Central-South-African (ECSA), Asian genotypes and Indian Ocean lineage [1]*.* In Senegal, gallery forest mosquitoes maintain a sylvatic transmission cycle where sporadic cases or small outbreaks can occur among humans living in rural areas, while the virus is mainly transmitted by *Aedes aegypti* in urban settlements [2,3]. Like in other African and Asian countries, a recurring CHIKV resurgence was observed in populations lacking herd immunity [4]*.* In Senegal, CHIKV outbreaks follow a cyclic pattern in rural regions, reemerging after a period of silence, usually every decade. This pattern aligns with the turnover time needed for susceptible animal hosts, mainly non-human primates [5,6]. After the first isolation from a bat in 1962, the first human infection was reported in Dakar region in 1966 [7] before the first outbreak in 1982 [8]. Later on, several investigations in the western part of the country demonstrated a high seroprevalence rate among whole blood donors in 1996 in Kaffrine as well as acute infections in several health workers from Niakhar in 1997 [9]. More recently in 2006, a cluster of foreign travellers were found infected by the virus after a stay in the neighbourhood of Dakar [10]. Until 1990s, CHIKV was not considered as a public health concern in Southeastern Senegal [11]. However, in-depth studies undertaken in 2000s highlighted the occurrence of zoonotic amplification and sporadic outbreaks [6], the most recent ones being in the Kedougou region [2,12].

Kedougou is a major arbovirus hotspot as highlighted by an extensive surveillance through a nationwide syndromic network and a passive surveillance in public health structures coordinated by the Ministry of Health and the Institut Pasteur de Dakar (IPD) [13] as well as a long-term entomological surveillance [3]. Moreover, the region has recently experienced an economic boom with the intensification of gold mining activity in rural areas, causing migration of human populations from various horizons and subsequent environment changes with an increased risk of pathogens exposition.

Here we carry out the genomic characterization of the CHIKV strain of an ongoing epidemic in Kedougou in 2023.

## Materials and methods

### Human and mosquito sample collection, and molecular diagnostic assays

Whole blood samples from suspected cases identified during the investigations were collected in EDTA tubes in the different healthcare sites or at the residence of suspected cases, stored in a cool box with ice, and sent on a daily basis to the IPD station in Kedougou for CHIKV molecular diagnostic as previously described [2] before weekly transmission to the WHO collaborating Center for Arboviruses and Hemorrhagic Fever Viruses (CRORA) in IPD in Dakar for complementary laboratory analysis [12]. The clinical criteria pinpointed suspected cases displaying fever along with two or more symptoms like headache, retro-orbital pain, arthralgia, myalgia, nausea/vomiting, or rash. Door-to-door epidemiological investigations targeted individuals who lived or resided in an epidemic area from 1 June 2023 until the investigation date. Confirmed cases fulfilled the clinical criteria and were confirmed via laboratory testing (positive IgM serology, virus detection by PCR). Meanwhile, mosquitoes collected from the entomological investigations in Kedougou were also tested onsite for CHIKV molecular detection.

### Next generation sequencing

Samples from confirmed cases with acute infection were processed to obtain the whole genome by a target enrichment standard hybridization workflow using the Twist Biosciences Comprehensive Viral Research Panel (CVRP), comprising approximately one million 120-base pair probes designed to target 15,488 distinct viral strains that infect both humans and animals, as previously described [14]. Briefly, extracted RNA samples were used as a template for a reverse transcription step with the SuperScript IV Reverse Transcriptase kit (Invitrogen, Thermo Fisher, USA) followed by a DNA fragmentation, telomere repair, dA-Tailing and a ligation with Universal Twist adapters before a final libraries amplification. A single pooled library was finally prepared from the indexed library-prepped samples before hybridization of the targets in solution, then binding of hybridized targets to desired streptavidin beads. Enriched sample libraries obtained as recommended by Twist Technical Support were loaded onto an Illumina iSeq 100 sequencing system as recommended by the manufacturer. Since no CHIKV sequence from the Kedougou 2015 outbreak was available, eight isolates obtained from the CRORA biobank were sequenced for further analysis.

### Data analysis

**Figure 1.**
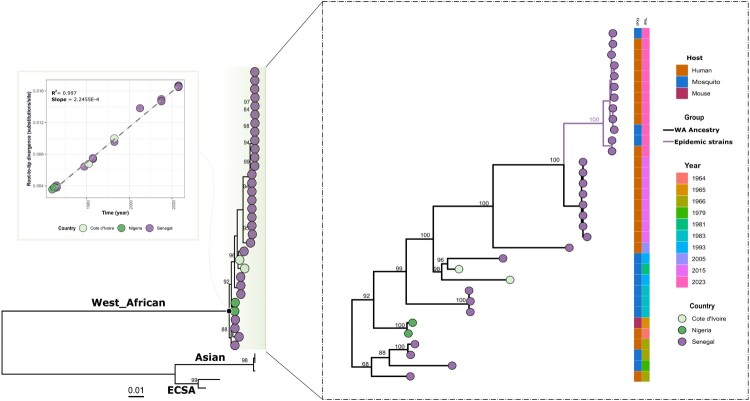
Time-scaled phylogenetic tree of sequenced CHIKV strains sampled during Kédougou outbreak in 2023. The tree was constructed using nearly complete genomes of CHIKV. Parent node of the outbreak sequences is annotated with tMRCA and 95% HPD interval of node age.

Raw data were collected in fastq format and the genome assembly was performed using the CZ-ID platform, an open-source metagenomics tool available at http://czid.org [15]. Default threshold filters were applied for reads Quality Check, base-calling, and consensus generation. A minimum depth of coverage of 10X was used for base calling. All generated sequences during this work were aligned with a representative dataset of available CHIKV sequences (Appendix 1) using MAFFT with default parameters and a maximum likelihood tree was subsequently performed using IQ-TREE with the best model determined by ModelFinder (MF) as previously described [7]. In parallel a second 1000 iterations-based ML tree built with only WA genotype strains. MF was used again to analyse the WA genotypes sequences dataset that was used as input for the root-to-tip analysis using Tempest [16]. Bayesian phylogenetic trees, calibrated over time, were constructed to estimate the emergence date (time to the most recent common ancestor, TMRCA) of the transmission lineage circulating during the 2023 outbreak. Analysis was performed based on nearly complete genomes using BEAST v.1.10.4 bayesian skygrid model as previously described [17]. tMRCA and 95% HPD interval information related to the emerging 2023 CHIKV clade were added as caption (Figure 1).

Amino acid (aa) changes spanning coding region of newly generated CHIKV sequences against human sequence obtained in 2005 in Kedougou (HM045817) were called using a python script according to a previously described protocol [18]. Obtained aa changes are organized by gene and observed frequencies of each aa change in 2015 and 2023 strains were also provided (Table 1).

**Table 1.** Non-synonymous mutations were observed in both structural and nonstructural genes in Chikungunya virus strains from 2015 and 2023 in Kedougou.

Proteins class	Gene	Mutation	Mutation frequency	
			2015 Strains	2023 Strains
Non-structural proteins	NSP1	P57S**	0%	100%
		I313L***	100%	100%
	NSP2	V153A*	100%	0%
		S259T	0%	16.67%
		V565A***	100%	100%
		N580S***	100%	100%
		T648N	37.50%	0%
		A674V	0%	58.33%
	NSP3	T192I***	100%	100%
		P367L*	100%	0%
		N385S***	100%	100%
		E438V***	100%	100%
		L461P***	100%	100%
		T509I	12.50%	0%
	NSP4	T72M***	100%	100%
		A462T	0%	8.33%
		S591C***	100%	100%
		I603V**	0%	100%
Structural proteins	C	A55T*	100%	0%
		N79K*	100%	0%
		P92L*	100%	0%
		I167V	0%	8.33%
	E3	–	–	–
	E2	M92T***	100%	100%
		H147Q	0%	16.67%
		S380T***	100%	100%
	6K	–	–	–
	E1	I280V	0%	8.33%
		V342A***	100%	100%

Note: CHIKV genome from 2005 in Kedougou (HM045817) was taken as reference.

*Unique to 2015 Strains.

**Unique to 2023 Strains.

***Shared by 2015 and 2023 Strains.

## Results and discussion

On early August 2023, a cluster of five Chikungunya virus-infected patients in the Kedougou region were identified by one-step RT-qPCR assay [2]. Following the increasing number of cases, an investigation team of both MoH epidemiologists and an IPD multidisciplinary group was mobilized to cover the three health districts of the region (Saraya, Salemata and Kedougou). At the time of writing, more than 200 confirmed cases were reported in the region and the outbreak is expanding to other regions (Communication from the IPD Public Health Direction). The most common symptoms were fever, headaches and myalgia with no severe manifestation (personal communications, manuscript in preparation). Epidemiological investigations showed that suspected cases did not have any travel history outside the Kedougou region during the last two months prior outbreak report (data not shown, manuscript in preparation).

Nine human and three mosquito samples among the positive samples from the ongoing outbreak as well as eight isolates from the previous emergence in 2015 in Kedougou were selected for genomic characterization. The sequencing strategy employed enabled the acquisition of 12 nearly complete genomes, with coverage levels varying from 93.6% to 99.1%. The mean depth coverage achieved was 12,406X, with a range of 56 to 46538X, as detailed in Appendix 2.

**Figure 2.**
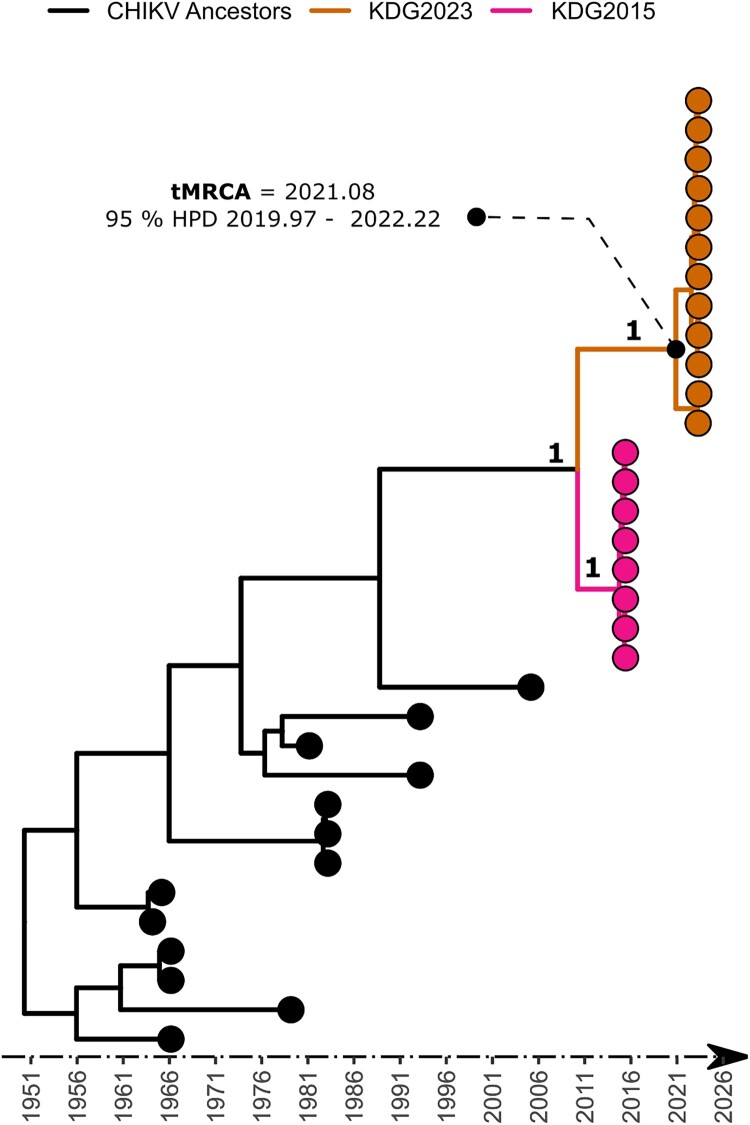
Phylogenetic relationship of newly generated CHIKV sequences and those previously available. Tree was annotated with country, year of sampling and host of West African genotype viral sequences. Only bootstrap values above 70 were represented on corresponding branches. Root-to-tip regression and time-scaled phylogenetic analysis show linear evolution between newly generated Chikungunya virus sequences obtained from the ongoing outbreak in Senegal 2023 and those from 2015 in Kedougou and the other West African genotype sequences.

The phylogenetic tree showed that viral strain from Kedougou 2023 groups with previous CHIKV from the WA genotype was identified during the more recent outbreaks in Kedougou in 2005 and 2015 (Figure 2). Indeed, blast analysis showed that the circulating strains in 2015 and 2023 exhibited mean nucleotide identity of 98.96% and 98.80% respectively with HM045817 strain obtained from humans in Kedougou in 2005 (Appendix 2). The Bayesian analysis conducted in this study demonstrates that the 2023 outbreak sequences form a monophyletic group, supported by a high posterior value of 1. The estimated time to the tMRCA suggests that the epidemic 2023 Chikungunya virus (CHIKV) strains likely emerged in early 2021, with a 95% highest posterior density (HPD) interval ranging from 2019.97 to 2022.22. This observation aligns with the sporadic serological cases reported in Kedougou in 2021 and 2022, although the data for these cases was not shown. Interestingly, the analysis included strains from the 2015 outbreak, which may have helped refine the results. This finding could aid in conducting in-depth investigations into the viral population dynamics between the 2015 and 2023 outbreaks in the future. Furthermore, the results suggest that the virus emerged at least approximately 1.5 years before being detected in humans during the 2023 outbreak. Given the robust human surveillance programme in Kedougou, this raises the possibility of a cryptic epizootic viral circulation, as previously reported in other studies [12]. Previous works have highlighted that sylvatic circulation can precede epidemic amplifications. These findings underscore the importance of implementing a One Health surveillance programme to mitigate virus spillover into human populations and establish early control strategies [5].

The polyprotein molecular analysis demonstrated 28 amino acids (aa) changes in both structural and nonstructural genes among either strains from CHIKV 2015 and CHIKV 2023 outbreaks in Kedougou (Table 1).

CHIKV 2015 and CHIKV 2023 polyproteins shared 12 aa substitutions compared to the older CHIKV 2005. Most of them are novel mutations identified in E1, E2 and nsP1-4, with E2-M92 T, E2-S380 T and nsP2-N580S in sites previously associated with type I Interferon modulation, antigenicity and host receptors binding [19,20]. CHIKV 2023 harbours eight unique mutations not seen in the CHIKV 2005 and 2015 strains, among which only two were consistently found in nonstructural proteins in all the generated sequences. Indeed, nsP1-P57S was found in the N-terminus (NT) methyltransferase and guanylyltransferase domain involved in methylation and capping of the newly synthesized RNA [21], while nsP4-I603 V was noted in the gene encoding the RNA-dependent RNA polymerase responsible for replicating viral RNA [22]. The six other CHIKV 2023-specific aa mutations (C-I167 V, E1-I280 V, E2-H147Q, nsP1-S259 T, nsP2-A674 V and nsP4-A462 T) occurred with lower frequency (8–59%) as shown in Table 1, and could then be either transient deleterious mutations which will be purged by purifying selection or potential new major variants whose prevalence is expected to increase as the virus expands.

Overall, the genomic analysis of our study reveals that the ongoing CHIKV epidemic in Kedougou in 2023 can be caused either by the introduction of a new WA CHIKV strain from other locations where undetected outbreaks occurred or by the re-emergence of an endemic WA genotype strain having evolved linearly (*R* = 0.99) (Figure 1) in a sylvatic in Kedougou context before a spillover event in the rural domain. Indeed, both scenarios are plausible because of (i) Kedougou ecological suitability for arbovirus circulation (favourable climate and abundant biodiversity), and (ii) the region economic growth leading to significant gatherings of human populations from diverse backgrounds, thereby increasing the risk of pathogen introductions.

## Conclusion

Our genomic characterization work demonstrates the endemicity of CHIKV in eastern Senegal and the constant threat it poses in terms of public health with cyclic resurgences. Indeed, the pathogen responsible for the 2023 epidemic arises from the regular molecular evolution of the strains from the 2005 and 2015 outbreaks. This supports previous work which highlights that the particular ecology of the Kedougou region, where human settlements overlap with the wild environment, allows the maintenance of the virus via potential non-human reservoirs and sylvatic vectors before spillover events [5,6]. Nonetheless, it is crucial not to overlook the potential for the strain to have been introduced from another infected area to Kedougou, underscoring the broad distribution and propagation of the WA genotype within the subregion.

Given that the main vectors during this epidemic were sylvatic Aedes species (data not shown), it is of interest to evaluate the impact of the different aa substitutions in the virus adaptation to the mosquito species in the region, similarly to what was done with the A226 V amino acid adaptative mutation in the E1 envelope glycoprotein of the ECSA genotype to *Ae. albopictus* in 2005 [23].

If the contemporary strains presented common features with the causative agents of the previous outbreaks, identification of the phenotypic association with the CHIKV 2023-specific aa changes requires more genomic, epidemiological and experimental data.

## Supplementary Material

Supplemental Material

Supplemental Material

## Appendices

### Appendix 1. Genbank accession numbers

**Table:**

Accession number	Year of isolation	Country
HM045817	2005	Senegal
AY726732	1983	Senegal
HM045785	1966	Senegal
HM045786	1964	Nigeria
HM045798	1966	Senegal
HM045807	1965	Nigeria
HM045815	1979	Senegal
HM045816	1966	Senegal
HM045818	1981	Cote d’Ivoire
HM045819	1993	Senegal
HM045820	1993	Cote d’Ivoire
KX262986	1983	Senegal
KX262995	1983	Senegal
KJ689453	2013	Micronesia
KJ451623	2013	Micronesia
KY435477	2014	Guyana
KY038946	1975	Central African Republic
KY704954	2016	Brazil

### Appendix 2. BLAST hit table of Chikungunya virus (CHIKV) sequences from 2015 and 2023 vs CHIKV 2005 (HM045817) and sequencing metrics

**Table:**

Sequence identity	Host	Max score	Total score	Query cover	E value	Percentage identity	Accession length (nt)	Num. reads	Depth (X)	Coverage (%)
412399	Mosquito	19648	19648	94%	0.0	98.81	11,714	944233	336	99
412625	Mosquito	19647	19647	94%	0.0	98.81	11,702	61266	829	98.9
412720	Mosquito	19551	19551	94%	0.0	98.71	11,595	334482	3459	98
417794	Human	19647	19647	94%	0.0	98.81	11,702	1523545	16782	98.9
417824	Human	19647	19647	94%	0.0	98.81	11,074	5004	56	93.6
425505	Human	19656	19656	94%	0.0	98.82	11,725	157608	2177	99.1
425541	Human	19647	19647	94%	0.0	98.81	11,713	5449	77	99
425581	Human	19651	19651	94%	0.0	98.82	11,713	299183	4221	99
425590	Human	19651	19651	94%	0.0	98.82	11,725	2481764	35524	99.1
425648	Human	19647	19647	94%	0.0	98.81	11,713	2532757	36006	99
425657	Human	19647	19647	94%	0.0	98.81	11,725	199809	2870	99.1
425797	Human	19651	19651	94%	0.0	98.82	11,713	3307797	46538	99
**Mean per cent identity against HM045817.1**						**98.80**				
