# Supplementary material for: Genomic characterization of a reemerging Chikungunya outbreak in Kedougou, Southeastern Senegal, 2023
Source: Emerg Microbes Infect. 2024 Jun 27;13(1):2373308. doi: 10.1080/22221751.2024.2373308 (PMC11268258; doi:10.1080/22221751.2024.2373308)
Supplement: Supplemental Material [file TEMI_A_2373308_SM1127.docx]

Appedix1 : Genbank accession numbers.

| Accession number | Year of isolation | Country |
| --- | --- | --- |
| HM045817 | 2005 | Senegal |
| AY726732 | 1983 | Senegal |
| HM045785 | 1966 | Senegal |
| HM045786 | 1964 | Nigeria |
| HM045798 | 1966 | Senegal |
| HM045807 | 1965 | Nigeria |
| HM045815 | 1979 | Senegal |
| HM045816 | 1966 | Senegal |
| HM045818 | 1981 | Cote d’Ivoire |
| HM045819 | 1993 | Senegal |
| HM045820 | 1993 | Cote d’Ivoire |
| KX262986 | 1983 | Senegal |
| KX262995 | 1983 | Senegal |
| KJ689453 | 2013 | Micronesia |
| KJ451623 | 2013 | Micronesia |
| KY435477 | 2014 | Guyana |
| KY038946 | 1975 | Central African Republic |
| KY704954 | 2016 | Brazil |
