# Supplementary material for: Genomic characterization of a reemerging Chikungunya outbreak in Kedougou, Southeastern Senegal, 2023
Source: Emerg Microbes Infect. 2024 Jun 27;13(1):2373308. doi: 10.1080/22221751.2024.2373308 (PMC11268258; doi:10.1080/22221751.2024.2373308)
Supplement: Supplemental Material [file TEMI_A_2373308_SM1122.docx]

Appendix 2 : BLAST Hit Table of Chikungunya virus (CHIKV) sequences from 2015 and 2023 vs CHIKV 2005 (HM045817) and sequencing metrics.

| Sequence Identity | Host | Max Score | Total Score | Query Cover | E value | Percentage identity | Accession Length (nt) | Num. Reads | Depth (X) | Coverage (%) |
| --- | --- | --- | --- | --- | --- | --- | --- | --- | --- | --- |
| 412399 | Mosquito | 19648 | 19648 | 94% | 0.0 | 98.81 | 11,714 | 944233 | 336 | 99 |
| 412625 | Mosquito | 19647 | 19647 | 94% | 0.0 | 98.81 | 11,702 | 61266 | 829 | 98.9 |
| 412720 | Mosquito | 19551 | 19551 | 94% | 0.0 | 98.71 | 11,595 | 334482 | 3459 | 98 |
| 417794 | Human | 19647 | 19647 | 94% | 0.0 | 98.81 | 11,702 | 1523545 | 16782 | 98.9 |
| 417824 | Human | 19647 | 19647 | 94% | 0.0 | 98.81 | 11,074 | 5004 | 56 | 93.6 |
| 425505 | Human | 19656 | 19656 | 94% | 0.0 | 98.82 | 11,725 | 157608 | 2177 | 99.1 |
| 425541 | Human | 19647 | 19647 | 94% | 0.0 | 98.81 | 11,713 | 5449 | 77 | 99 |
| 425581 | Human | 19651 | 19651 | 94% | 0.0 | 98.82 | 11,713 | 299183 | 4221 | 99 |
| 425590 | Human | 19651 | 19651 | 94% | 0.0 | 98.82 | 11,725 | 2481764 | 35524 | 99.1 |
| 425648 | Human | 19647 | 19647 | 94% | 0.0 | 98.81 | 11,713 | 2532757 | 36006 | 99 |
| 425657 | Human | 19647 | 19647 | 94% | 0.0 | 98.81 | 11,725 | 199809 | 2870 | 99.1 |
| 425797 | Human | 19651 | 19651 | 94% | 0.0 | 98.82 | 11,713 | 3307797 | 46538 | 99 |
| **Mean percent identity against HM045817.1** | | | | | | **98.80** |  |  |  |  |
